# Niagara Falls

**Published:** 1881-09

**Authors:** 


### NIAGARA FALLS.

WE frequently read in the newspapers about the extortions that are practiced on visitors to Niagara Falls; but we seldom notice any suggestions to visitors that will enable them to escape these exactions and swindles; but it is very easily done. With characteristic enterprise, owners of property on the American side have fenced in their lands, so that it is difficult to get a view of the Falls from this side without paying gate money—50 cents—at various points along the river. These, with carriage hire and other systematic methods of plunder, are calculated to reach the bottom of the average pocket, and leave one nothing with which to cross the river.

I have been an occasional visitor to Niagara Falls during thirty years, and have lived for years within hearing of their perpetual roar; but years before obstructions were placed along the river, I never considered that I had seen the Falls till I got on the Canada side, and I never yet visited Goat Island, as I had no desire to tramp over ground the whole of which I could see as a map spread out before me from the Canadian side. The principal Fall—the Horse-Shoe—is altogether in Canada, and cannot be seen to advantage on the American side. The American Fall, which is much the smaller, is formed from the water that flows along the American side of Goat Island, and as there is a curve in that part of the river, you look directly on its face from the Canada side.

Whenever I visit the Falls, on leaving the railroad depot I turn a deaf ear to the solicitations of hack drivers and venders of curiosities, and walk to the foot-bridge below the Falls. The toll is 25 cents. I cannot afford to ride across the bridge, for I should lose the finest views to be anywhere obtained. I take my time, going leisurely along and making frequent stops on the bridge. On the Canada side there is a sense of freedom—a relief from many nuisances. There are fewer hackmen here, and they are less obtrusive, and there can be no obstructions along the river, for the Government of Canada owns the military road, 100 feet wide, the whole length of the river along its banks. No attempts have been made to change the face of nature, and you see the Falls and the river banks as they were ages ago. The hotels are fully as good as on the American side, it less pretentious, and their charges are quite as reasonable. A carriage can be hired for a reasonable sum to take you to the Railroad Bridge and

Whirlpool, two miles below, and then one can walk or ride across to the depot on the other side. As to the Whirlpool, a great deal of the *whirl* is in the imagination, like many other objects at the Falls. You get all there really is of Niagara Falls by walking across the foot-bridge and then up to the Horseshoe Falls, leisurely, getting different views as you pass along. To do this well requires two to three hours, and all other sights and entertainments draw largely on the imagination, and are a vexation of spirit beside a useless expense.

By all means secure a good breakfast or a good dinner before starting out. Sight-seeing on an empty stomach loses half its interest.

